# PROTOCOL: In‐person interventions to reduce social isolation and loneliness: An evidence and gap map

**DOI:** 10.1002/cl2.1340

**Published:** 2023-06-22

**Authors:** Vivian Welch, Elizabeth Tanjong Ghogomu, Sierra Dowling, Wan Yuen Choo, Raudah M. Yunus, Tengku A. M. Tengku Mohd, Niobe Haitas, Sivan Bomze, Simone Dahrouge, Edward Garcia, Julianne Holt‐Lunstad, Mathias Lasgaard, Michelle H. Lim, Kate Mulligan, Douglas M. Salzwedel, Pamela Qualter, Paul C. Hébert, Christopher Mikton

**Affiliations:** ^1^ Bruyère Research Institute Ottawa Canada; ^2^ Bruyère Research Institute University of Ottawa Ottawa Canada; ^3^ Department of Social & Preventive Medicine, Faculty of Medicine University of Malaya Kuala Lumpur Malaysia; ^4^ Public Health Medicine Universiti Teknologi MARA Sungai Buloh Malaysia; ^5^ Universiti Sains Islam Malaysia, Faculty of Medicine and Health Sciences Putra Nilai Malaysia; ^6^ Canadian Red Cross Montreal Canada; ^7^ Canadian Red Cross Mississauga Canada; ^8^ Canadian Red Cross Mississauga Canada; ^9^ Foundation for Social Connection Washington District of Columbia USA; ^10^ Brigham Young University Provo Utah USA; ^11^ DEFACTUM ‐ Public Health and Health Services Research Central Denmark Region Aarhus N Denmark; ^12^ University of Sydney, School of Public Health Sydney Australia; ^13^ Ending Loneliness Together Melbourne Australia; ^14^ University of Toronto, Dalla Lana School of Public Health Toronto Canada; ^15^ Department of Anesthesiology, Pharmacology and Therapeutics University of British Columbia Vancouver Canada; ^16^ University of Manchester, Mancehster Institute of Education Manchester UK; ^17^ Bruyere Research Institute Palliative Care University of Ottawa Ottawa Canada; ^18^ World Health Organization, Department of Social Determinants of Health Geneva Switzerland

## Abstract

This is the protocol for an evidence and gap map. The objectives are as follows: This EGM aims to map available evidence on the effects of in‐person interventions to reduce social isolation and/or loneliness across all age groups in all settings.

## 1 BACKGROUND

### 1.1 Introduction

#### 1.1.1 The problem, condition, or issue

Social isolation is the objective lack or paucity of social contact and infrequent interactions with others (Badcock et al., 2022; Donovan et al., 2020; Leigh‐Hunt et al., 2017). Loneliness is a related concept defined as the subjective, negative feeling of inadequate meaningful connections resulting from an unmet need or discrepancy between desired and actual social relationships (Perlman et al., 1981; Prohaska et al., 2020). Loneliness has two components: an emotional component (unpleasant, negative feeling) and a social cognition component (perception of social disconnection from other people with a desire to be connected) (Badcock et al., 2022). Loneliness can also be a transient normal experience or chronic with negative physical and mental health consequences (Akhter‐Khan et al., 2020; Qualter et al., 2015).

The two concepts, social isolation, and loneliness, are distinct; social isolation is objective and associated with social contact while loneliness is subjective and related to social connectedness (O'Rourke et al., 2018). One may occur without the other, although they are related and may also co‐occur. When social isolation and loneliness co‐occur, the risk of mortality is exacerbated (Beller & Wagner, 2018). People may have a social network and feel lonely, while some with a small network may not.

Social isolation and loneliness can occur across all age groups and are associated with serious health consequences including anxiety and depression, cardiovascular disease, and premature mortality (Cené et al., 2022; Leigh‐Hunt et al., 2017). Poor relationships are associated with 32% increased risk of stroke, 29% increased risk of coronary heart disease (Cené et al., 2022; Valtorta et al., 2016), and 26% increased risk of all‐cause mortality (Donovan et al., 2020; Holt‐Lunstad et al., 2015). Incident depression and dementia have a bi‐directional relationship with both social isolation and loneliness (Cené et al., 2022; Donovan et al., 2020) although several studies have reported that dementia is associated with loneliness than social isolation (Cené et al., 2022; Holwerda et al., 2014; Rafnsson et al., 2020). Both social isolation and loneliness are associated with negative health‐related behaviors such as smoking and physical inactivity (Cené et al., 2022; Menec et al., 2020). The negative health impacts of social isolation and loneliness have been shown to increase health and social care service use (Cotterell et al., 2018; Windle et al., 2012). These negative impacts occur when contextual and risk factors affecting social relationships persist and individuals do not use appropriate coping strategies to address them (Akhter‐Khan et al., 2022; Elder et al., 2012).

Since the onset of the COVID‐19 pandemic, movement restriction policies have made social isolation and loneliness prominent global issues and a public health priority (Galvez‐Hernandez et al., 2022; Holt‐Lunstad, 2022; WHO, 2021). The prevalence of severe loneliness increased by 15% and social isolation by 13% in adults 18 years or older across 101 countries during the pandemic (O'Sullivan et al., 2021). Small increases in the prevalence of loneliness were also observed in a recent synthesis of longitudinal studies during the COVID‐19 pandemic (Ernst et al., 2022). The prevalence is hard to measure across the lifespan because of the lack of standardized measurement instruments and definitions, and the use of different cut‐off points and age categories (Holt‐Lunstad, 2022; Prohaska et al., 2020). A recent systematic review and meta‐analysis on the prevalence of loneliness pre‐COVID‐19 pandemic across 113 countries (Surkalim et al., 2022) showed varying rates for adolescents (9.2%–14.4%), young adults (1.8%–9.4%), middle‐aged adults (2.4%–12%), and older adults (4.2%–24.2%) depending on the country. A prevalence study in a population‐based adult cohort showed that social isolation increases with age from 5.4% (95% confidence interval [CI]: 4.7 to 6.0) in the youngest age group (18–39 years) to 21.7% (95% CI: 19.5 to 24.0) in the oldest age group (70–79 years) (Röhr et al., 2021). The global prevalence of social isolation in community‐dwelling older adults was found to be 25% (95% CI: 21 to 30) (Teo et al., 2022). Most of the studies were conducted in high income countries, especially in Europe, with very few in low‐middle‐income countries (Fakoya et al., 2020; Surkalim et al., 2022).

Both social isolation and loneliness are linked to less social support and can be triggered by situational factors such as adversity, significant life changes or transitions, such as moving away from home, starting a new job, becoming a new parent, illness, and the death of a spouse or parent (Badcock et al., 2022; Elder et al., 2012; Lim et al., 2020; Qualter et al., 2022). They are associated with risk factors including individual factors (e.g., personality, maladaptive cognition, poor health, disability or mobility impairment, cognitive impairment), interpersonal or social factors (e.g., peer victimization or discrimination, poor relationship quality, quantity of friends or social contacts, living alone), socio‐environmental factors (e.g., neighborhood deprivation, inaccessible location of residence, housing, cultural prejudice), and demographic factors (e.g., age, gender, educational level, low socio‐economic status, unemployment) (Badcock et al., 2022; Elder et al., 2012; Lim et al., 2020; O'Sullivan et al., 2021; Qualter et al., 2022).

Many systematic reviews have evaluated the effectiveness of interventions to reduce social isolation or loneliness with conflicting findings demonstrating a need for better quality research (Masi et al., 2011; Victor et al., 2018; Williams et al., 2021). A number have focused on older adults, but social isolation and loneliness affect people across the life span, including young people (Qualter et al., 2015; Surkalim et al., 2022), with interventions designed specifically for them (Eccles et al., 2021). Most of the reviews have focused on people living in the community or long‐term care settings (Fakoya et al., 2020; Grenade et al., 2008). There is limited research addressing social isolation and/or loneliness for patients in clinical settings (NASEM, 2020). Studies that consider hospitalized patients focus on screening and detection of loneliness and social isolation, the impact of social isolation and loneliness on health service use and which interventions may be used rather than the assessment of the effectiveness of interventions to reduce social isolation and loneliness (Grenade et al., 2008; NASEM, 2020; Proffitt et al., 1993; Razai et al., 2020; Zamir et al., 2018).

The impact of interventions has been found to differ depending on population characteristics such as coping skills, needs, degree of loneliness, and contextual factors like age, socioeconomic status, health condition, and place of residence (Fakoya et al., 2020). Therefore, there is no one‐size‐fits‐all approach, and it is important to tailor appropriate interventions to individuals’ needs and contexts (Akhter‐Khan et al., 2020; Fakoya et al., 2020; Mann et al., 2017).

There are health equity issues related to social isolation and/or loneliness such as the gap in evidence from low‐middle income countries (Surkalim et al., 2022), limited access to interventions caused by disabilities and lack of transportation, or limited programs in rural areas compared to urban areas (Dassieu et al., 2021; NASEM, 2020; Qualter et al., 2022). Social isolation and/or loneliness related to structural inequities (e.g., intersectional discrimination across race, gender, socioeconomic status; age‐based discrimination and ethnic minorities), have a negative impact on health outcomes (Dassieu et al., 2021).

This current evidence and gap map will identify areas where evidence is available, as well as any gaps in research related to in‐person interventions for social isolation and loneliness across any age.

#### 1.1.2 The intervention

Different types of interventions for reducing social isolation (Dickens et al., 2011; Findlay, 2003), loneliness (Cohen‐Mansfield et al., 2015; Eccles et al., 2021; Hagan et al., 2014; Jarvis et al., 2020; Mann et al., 2017; Masi et al., 2011; Veronese et al., 2021), or both social isolation and loneliness (Cattan et al., 2005; Gardiner et al., 2018; Poscia et al., 2018) have been described and assessed in several systematic and scoping reviews. However, there is a lack of a standardized framework for describing these interventions (Fakoya et al., 2020; Prohaska et al., 2020). Interventions for reducing social isolation and loneliness are often complex with multiple and interacting components, working through different potential mechanisms of action (Fakoya et al., 2020; Gardiner et al., 2018).

Several approaches have been used to categorize interventions in some reviews. The interventions have been categorized by the format or delivery mode or type as one‐on‐one or group‐based (Cohen‐Mansfield et al., 2015; Dickens et al., 2011; Fakoya et al., 2020; Findlay, 2003; Hagan et al., 2014; Masi et al., 2011; Poscia et al., 2018), or technology or non‐technology (in‐person) based (Eccles et al., 2021; Masi et al., 2011). They have also been categorized by the type, or strategy, being classified as interventions for social skills training, enhancing social support, enhancing social interaction or social cognition training (Masi et al., 2011). Other terms have been used as a rationale for categorization, such as the focus, nature or goal of the intervention (Cohen‐Mansfield et al., 2015; Fakoya et al., 2020; Masi et al., 2011); the purpose, intended outcomes and mechanisms by which interventions target social isolation and loneliness (Fakoya et al., 2020; Gardiner et al., 2018).

The scoping review by Mann et al. classifies interventions as direct or indirect, and also articulates various levels of engagement for those delivering the interventions following the socio‐ecological model: individual level, relationship and community level, and societal level (Mann et al., 2017).

Another scoping review (O'Rourke et al., 2018) classified interventions for reducing loneliness by their components into nine types: one‐to‐one personal contact, activity group, animal contact, skills course interventions, or varied/non‐specific interventions, reminiscence, support group, model of care and public broadcast.

This evidence and gap map will focus on in‐person interventions that are non‐technology based and delivered face‐to‐face since there is a gap map on digital interventions for older adults (Welch et al., 2022b).

#### 1.1.3 Why it is important to develop the EGM

The existing body of evidence for interventions to mitigate social isolation and/or loneliness is characterized by small, low‐quality trials, with inconsistent terminology and conclusions on their effectiveness (Eccles et al., 2021; Fakoya et al., 2020; Prohaska et al., 2020; Veronese et al., 2021). With the rapid growth of evidence in this sector, this evidence and gap map will demonstrate areas where evidence is available and areas where there are gaps that researchers, decision and policymakers could use to help select interventions and prioritize future research. It will also improve the discoverability of evidence on different types of interventions and enhance their use for informed decision‐making by stakeholders including health and social care providers, policymakers, citizens, caregivers, and patients.

#### 1.1.4 Existing evidence and gap maps and/or relevant systematic reviews

There is rapidly expanding research on alleviating social isolation and/or loneliness since the COVID‐19 pandemic. Several relevant systematic reviews have been conducted and included in three umbrella reviews (Boulton et al., 2021; Jarvis et al., 2020; Veronese et al., 2021). Boulton et al. found mixed evidence of effectiveness on loneliness for remote befriending, social support, and low intensity psychosocial interventions. Jarvis assessed various interventions addressing loneliness in older adults and found limited effect, with the greatest effect in a social cognition intervention. Veronese et al. reported low or very low‐quality evidence of three interventions (meditation/mindfulness, social cognitive training and social support) that reduced loneliness. A scoping review of reviews showed the lack of studies conducted in low‐middle income countries (Fakoya et al., 2020).

One evidence and gap map has been conducted on remotely delivered digital interventions including befriending, social support, and low‐intensity psychosocial interventions for social isolation and loneliness in older adults (Boulton et al., 2021). It showed mostly low‐quality reviews and few studies on older people who are not caregivers or who do not have a particular chronic illness. Our group is currently working on another gap map on digital interventions for older adults with a broader scope of interventions and including caregivers (Welch et al., 2022; Welch et al., 2022b), but there is currently no mapping of evidence for in‐person interventions to reduce social isolation and loneliness across all ages.

## 2 OBJECTIVES

This EGM aims to map available evidence on the effects of in‐person interventions to reduce social isolation and/or loneliness across all age groups in all settings.

Specific objectives are as follows:

1. To identify existing evidence from primary studies and systematic reviews on the effects of in‐person interventions that are non‐technology based and delivered face‐to‐face to reduce social isolation and/or loneliness across all age groups.
2. To identify research evidence gaps for new high‐quality primary studies and systematic reviews.
3. To highlight evidence of health equity considerations from included primary studies and systematic reviews.

## 3 METHODS

We will follow the Campbell Collaboration guidance for producing an evidence and gap map (White et al., 2020).

### 3.1 Evidence and gap map: Definition and purpose

Evidence and gap maps are a systematic evidence synthesis product with a visual presentation of existing evidence relevant to a specific research question (Snilstveit et al., 2013; White et al., 2020). They display areas where evidence is available, areas where there are gaps in evidence, and the quality of existing evidence.

The evidence and gap map is typically a two‐dimensional matrix with interventions as row headings and outcomes as column headings (Snilstveit, 2016; White et al., 2020). The studies with evidence on the corresponding intervention and outcome are shown within each cell of the matrix. This map will identify areas of evidence and any gaps in research related to using in‐person interventions for social isolation and/or loneliness across all ages.

### 3.2 Framework development and scope

We developed an intervention‐outcome framework by adapting our conceptual framework from the digital interventions EGM (Welch et al., 2022). We expanded the non‐digital intervention and outcome categories to attain evidence‐based, clear and distinct categories that are practical and useful to a broad audience by using several existing frameworks, reports, and reviews.

In consultation with stakeholders, we identified and reviewed other frameworks including the framework described by Masi (Masi et al., 2011), the framework for the Campaign to End Loneliness by Jopling (Jopling, 2020), the framework by Mann (Mann et al., 2017), and the socio‐ecological framework adapted by the World Health Organization for strategies to reduce social isolation and loneliness (WHO, 2021), the framework for evidence‐based interventions for youth reporting loneliness (Qualter et al., 2022), and the social relationship expectations framework (Akhter‐Khan et al., 2022). We also considered the National Academies of Sciences, Engineering, and Medicine Consensus study report on social isolation and loneliness in older adults (NASEM, 2020), the American Association of Retired Persons (AARP) Foundation report on social isolation (Elder et al., 2012), the taxonomy to evaluate social isolation and loneliness interventions developed by the Foundation for Social Connection, and three reviews on loneliness (Holt‐Lunstad, 2022; Lim et al., 2020; Ogrin et al., 2021).

Six of the existing frameworks and reviews have adopted the socio‐ecological framework when considering interventions to reduce social isolation and/or loneliness (Holt‐Lunstad, 2022; Lim et al., 2020; Mann et al., 2017; Ogrin et al., 2021; Qualter et al., 2022; WHO, 2021). The socio‐ecological framework has been used to explore the complex dimensions of other public health issues like violence and abuse (CDC, 2015; WHO, 2002) and health promotion (Wold et al., 2018). The model examines the relationship between risk and protective factors at different levels of influence including individual, relationship, community, and societal levels. The individual level focuses on personal characteristics that increase risks. The relationship level focuses on risk factors involving close social relationships with family and friends. The community level explores risk factors from wider social relationships in community settings such as schools, workplaces, and neighborhoods. The societal level of influence considers broad societal risk factors such as policies and cultural norms.

Social isolation and loneliness are associated with the lack of meaningful social connections which can occur at any of these four levels (Holt‐Lunstad, 2018, Holt‐Lunstad, 2022; Lim et al., 2020; Ogrin et al., 2021). Risk factors for social isolation and loneliness can be co‐occurrent, inter‐related, and can operate at multiple levels (Holt‐Lunstad, 2022; Lim et al., 2020; Qualter et al., 2022). Interventions may target risk factors at multiple levels of the socio‐ecological model by creating and maintaining meaningful social connections or a combination of other mechanisms, such as changing negative social cognition or providing support to enhance social interactions.

We will consider non‐technology‐based interventions delivered in‐person to alleviate social isolation and/or loneliness across all age groups in all settings. None of the existing frameworks or taxonomies provide mutually exclusive categories and subcategories for classifying the interventions for this evidence and gap map. They all demonstrate the complexity, diversity, and interdependencies of contextual or risk factors, and mechanisms that shape social relationships.

We will therefore focus on delivery and classify interventions into five main categories based on who is providing the intervention and where it is provided: self delivery, interpersonal delivery, community‐based delivery, societal level delivery, and multi‐component or complex interventions.

Outcomes will be based on the level of impact of interventions. The impact of social isolation and loneliness interventions depend on how well they were implemented, therefore, we will consider both process indicators or implementation outcomes and other outcomes including health and psychosocial outcomes, indicators of social connections as well as cost and cost‐effectiveness outcomes (Jopling, 2020; Windle et al., 2012).

### 3.3 Stakeholder engagement

We established an Advisory Board of key stakeholders to contribute toward defining the scope and developing the framework for the map as well as interpreting the findings. They include academics, advocates, policy and decision‐makers, from relevant organizations (e.g., WHO, Canadian Red Cross, Global Initiative on Loneliness and Connection, US Foundation for Social Connection, and Ending Loneliness Together) who are involved in research and working to address social isolation and loneliness.

The Advisory Group met virtually on December 13 and 16, 2021 to discuss the scope of the evidence and gap map and existing frameworks that could be considered in developing the intervention‐outcome framework for this evidence gap map. They met again in June 2022 to provide feedback on the framework. They will be consulted to provide feedback on the revised framework and on the preliminary findings and draft map.

### 3.4 Conceptual framework

**Figure 1:**
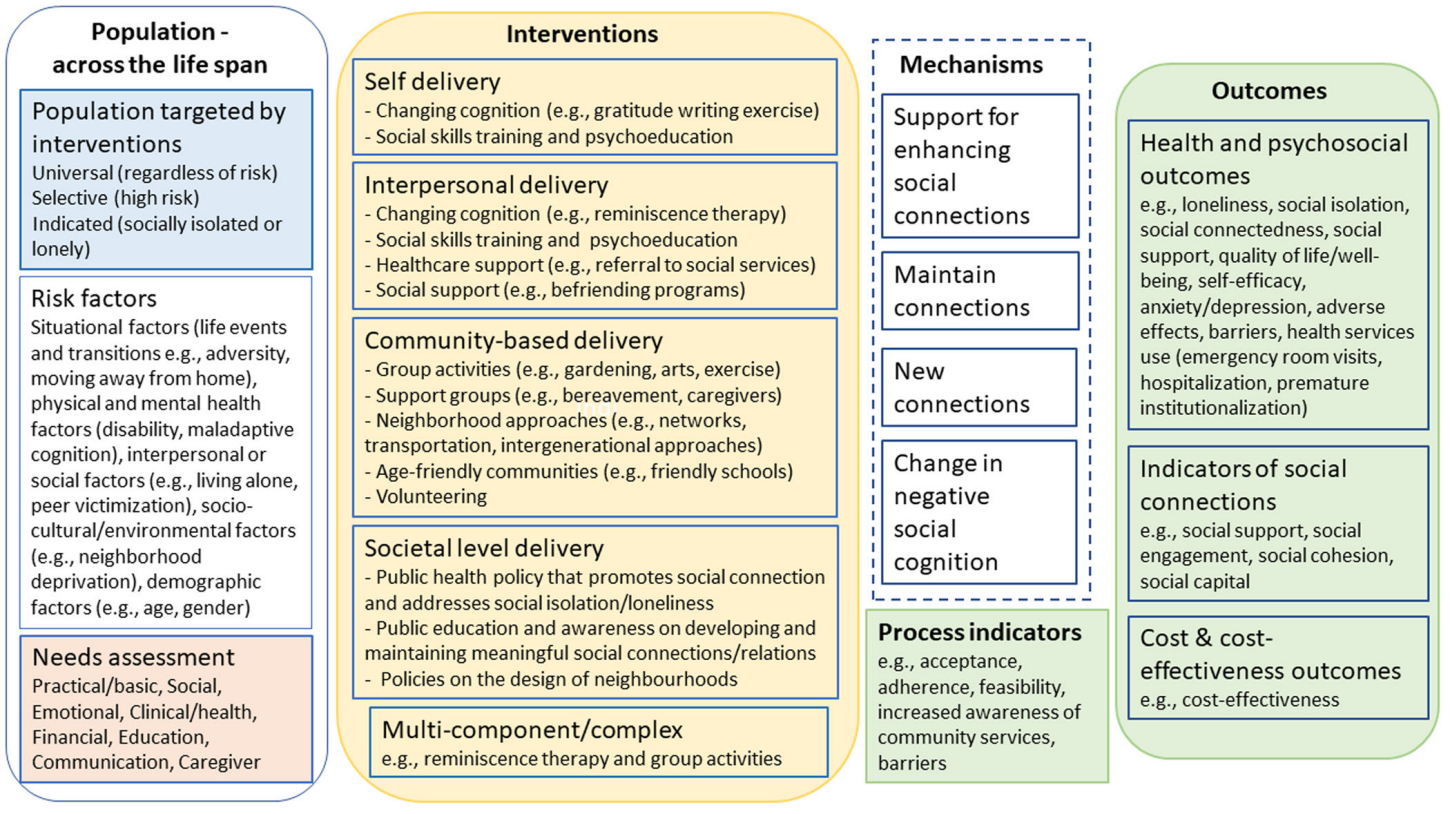
Conceptual framework.

The conceptual framework (Figure 1) considers possible pathways for interventions to bring about expected changes and outcomes based on the understanding of the population risk factors and needs that may trigger social isolation or loneliness. It is based on theoretical underpinnings with the following key components:

- population contexts, risk factors and needs that may trigger social isolation or loneliness.
- types of interventions required to address social isolation or loneliness,
- the mechanisms of change by which the interventions address social isolation or loneliness, and
- process indicators (e.g., acceptability) and outcomes (e.g., loneliness).

#### 3.4.1 Population targeted by interventions

Social isolation and loneliness are complex public health issues and their occurrence across the lifespan is influenced by individual contextual and risk factors, needs, expectations, and coping skills which are all inter‐related and influence relationship ties (Akhter‐Khan et al., 2022; Elder et al., 2012; Gardiner et al., 2018; O'Rourke et al., 2018; Qualter et al., 2022). Contextual and risk factors such as structural changes that may cause displacement (e.g., moving schools or wars), living situations (e.g., living alone or in a care facility such as orphanage, long‐term care home), resources (available activities or social supports) affect people's motives, expectations, coping skills, and social relationships. Coping skills and social supports may be a protective factor if they allow people to promote their wellbeing or resilience. On the other hand, inadequate coping skills and social supports may be a risk factor for social isolation or loneliness.

Based on a public health approach, interventions may target anyone regardless of risk (universal), or target subpopulations at high risk (selective) or socially isolated or lonely people (indicated) (Springer et al., 2007). Categorizing target populations into these three orders gives a clearer picture and understanding of whom to prioritize and how to allocate resources more efficiently.

#### 3.4.2 Risk factors

Social isolation or loneliness may be triggered in both young and old across the life span by situational factors such as significant life events or transitions (e.g., adversity, moving away from home, retirement, death of a spouse, friend or relative) and may be associated with risk factors including physical and mental health factors (e.g., poor health, maladaptive cognition or cognitive decline, disability or impaired mobility, personality), interpersonal or social factors (e.g., living alone, peer victimization, social contacts, relationship quality,), socio‐cultural or environmental factors (e.g., neighborhood deprivation, inaccessible location of residence, cultural individualism, social discrimination, and stigma) and demographic factors (e.g., age, gender, socio‐economic status) (Dahlberg et al., 2022; Elder et al., 2012; Lim et al., 2020; NASEM, 2020; Qualter et al., 2022).

#### 3.4.3 Needs assessment

Socially isolated and lonely individuals across the lifespan have unmet needs that can be due to low social support or disruption in social interactions with people at different levels—individual, group, community, or societal (Elder et al., 2012; Holt‐Lunstad, 2022; NASEM, 2020; WHO, 2021). These needs include basic needs (housing, nutrition and food security, and healthcare), social and emotional needs (social connections and companionship), financial, education (skills development and learning), communication, caregiver needs, home modifications and maintenance, domestic assistance, mobility, personal care, respite care and civic engagement (meaningfulness and status, the need for having a purpose in later life or being able to contribute usefully to society) (Abdi et al., 2019; Bedney et al., 2010; Bee et al., 2014; Eccles et al., 2021).

Social support is a major component of social connection and may be provided to meet different needs. Social support can take the forms of instrumental/tangible, informational, emotional or belonging support (Elder et al., 2012; Holt‐Lunstad, 2022; NASEM, 2020). It can be perceived, or actual support provided through social connections with other people and through different sectors including health, transportation, housing, work, nutrition, environment, education, leisure: arts and entertainment (Holt‐Lunstad, 2022).

Loneliness is also associated with social relationship expectations that are influenced by personal, social, cultural and historical contexts and include the availability of social contacts (proximity), feeling cared for and relying on others (support), feeling close, understood, and listened to (intimacy), sharing interests and enjoyable experiences (fun), having opportunities to contribute meaningfully (generativity) and feeling valued and actively included (respect) (Akhter‐Khan et al., 2022). A discrepancy between expected and actual social relationships will result in loneliness.

It is important to asess and understand individuals’ specific contexts, risks, expectations, and needs to tailor appropriate interventions to reduce social isolation or loneliness (Akhter‐Khan et al., 2022; Eccles et al., 2021; Fakoya et al., 2020; Jopling, 2020; Lim et al., 2020; Mann et al., 2017; NASEM, 2020).

#### 3.4.4 Interventions

Interventions to reduce social isolation and/or loneliness are more effective when targeted to the individual's specific experience and context (such as triggers, risk factors, and accessibility to resources) (Eccles et al., 2021; Fakoya et al., 2020; Prohaska et al., 2020; Veronese et al., 2021). Interventions aimed at reducing social isolation may have different components than those aimed at reducing loneliness (O'Rourke et al., 2018). The interventions can be delivered through various modes to bring about changes at different levels—individual, relationship, community, and societal levels (Holt‐Lunstad, 2022; Lim et al., 2020; Mann et al., 2017; Ogrin et al., 2021; WHO, 2021). In addition, one component of an intervention may target multiple factors (O'Rourke et al., 2018). It is therefore challenging to categorize interventions by the risk factors they target following the socio‐ecological framework or by their mechanisms. To have mutually exclusive categories and subcategories on the evidence map, we will classify interventions as follows, based on who is providing the intervention and where the intervention is provided.

Self‐delivery: These are self‐guided interventions that focus on addressing personal characteristics (biological and sociodemographic factors, e.g., socio‐economic status) that may trigger social isolation or loneliness, through strategies that change the attitudes, beliefs, and behaviors of individuals. The purpose of these interventions is to affect changes in an individual that would result in reductions in isolation and loneliness. They can be facilitated with training, guidance, or available resources from the healthcare professionals. Examples include self‐guided social cognitive interventions like cognitive behavioral therapy, mindfulness or reminiscence therapy.

Interpersonal delivery: These are interventions that focus on building close meaningful personal relationships with family and others in the community, for example, friends, colleagues, neighbors, volunteers. They can be delivered by healthcare or social care professionals, volunteers or other people in the individual's social network. The purpose/aim of these interventions is to affect changes in an individual or a specific relationship or network of relationships that would result in reductions in isolation and loneliness. They are accomplished through mechanisms including changing cognition, social skills training and psychoeducation, healthcare support and social support. Examples include cognitive behavioral therapy, family therapy, social prescribing or friendship enrichment program.

Community‐based delivery: These interventions are delivered by healthcare or social care professionals, community workers/volunteers or other people within the same community setting. They can be delivered through neighborhood organizations, community‐based healthcare and social services or facilities. They focus on addressing risk factors in social settings and increasing opportunities for social interactions with others such as connecting to community group activities or peer support groups; neighborhood approaches like networks, transportation, meals on wheels; intergenerational approaches. They may also provide a supportive environment or encourage participation by improving access to amenities within the community, for example, built environment, age‐friendly communities, and volunteering. These interventions can affect changes in an individual, a specific relationship or network of relationships, or changes in the community that would result in reductions in isolation and loneliness.

Societal level delivery: Focus on policies and laws that address societal risk factors like discrimination and stigma, socio‐economic inequality or may seek to change social norms that prevent social connection such as policies addressing housing, employment, transportation and the environment. These interventions affect change(s) in broader society, resulting in reductions in isolation and loneliness. Examples include public awareness campaigns, coalition and partnership initiatives, or family‐friendly policies.

Multi‐component/complex: These are combinations of multiple components within the interventions involving the same/different types of delivery modes in the same study.

#### 3.4.5 Mechanisms

Different pathways or mechanisms related to contextual or risk factors, motives, expectations, and coping skills have been identified through which interventions may reduce social isolation or loneliness. The interventions may improve and maintain existing relationships or enable people to create new connections (Akhter‐Khan et al., 2022; Jopling, 2020; Mann et al., 2017) by addressing contextual or risk factors (Akhter‐Khan et al., 2022; Lim et al., 2020; Ogrin et al., 2021). Some interventions aim to change one's outlook or negative social cognition (Akhter‐Khan et al., 2022; Jopling, 2020; Mann et al., 2017; Masi et al., 2011; Ogrin et al., 2021) while others provide support to enhance social interactions (Mann et al., 2017; Masi et al., 2011; Ogrin et al., 2021). Some interventions involve building skills, purposeful activity, or implementing a philosophy of care within a facility (Akhter‐Khan et al., 2022). Some interventions are complex and may address social isolation or loneliness through multiple or poorly specified mechanisms (Akhter‐Khan et al., 2022; Holt‐Lunstad, 2018; Lim et al., 2020).

#### 3.4.6 Process indicators and outcomes

The potential of interventions to reduce social isolation or loneliness have been assessed through their acceptability, adherence, and feasibility. These process indicators determine progress toward outcomes such as health and psychosocial outcomes (e.g., loneliness, social isolation, social connectedness), indicators of social connections (e.g., social support, social engagement, social cohesion), as well as cost and cost‐effectiveness outcomes. See Glossary of terms (Supporting Information: Appendix 1).

We will use this conceptual framework to define and code the intervention and outcome categories and subcategories for the two‐dimensional matrix in the evidence and gap map.

### 3.5 Dimensions

#### 3.5.1 Types of study design

We will include on‐going and completed systematic reviews and primary studies with any study design that has a control group: randomized controlled trials, non‐randomized studies including control before‐after, and statistical matching quasi‐experimental studies.

The inclusion of systematic reviews will be based on the population, intervention, comparison, outcome (PICO) framework and if they meet at least four of the five criteria of a systematic review as defined by Moher et al. (Moher et al., 2015). That is, they describe adequate search methods used to identify studies, eligibility criteria for selection of studies, methods of critical appraisal of included studies, sufficient details or characteristics of included studies, and synthesis or analysis of findings of the included studies.

Eligible quasi‐experimental designs include quasi‐randomized studies, regression discontinuity designs, natural experiments, non‐equivalent comparison group designs and interrupted series designs with at least three data points before and after a discrete intervention (Waddington et al., 2014).

We will exclude any study designs with no control group such as longitudinal cohort studies and cross‐sectional studies, and those studies with interrupted time series designs with less than six data points.

We will include mixed methods studies, but exclusive qualitative research will be excluded.

We will include studies irrespective of their publication status.

#### 3.5.2 Types of intervention/problem

We will include any intervention which aims to reduce social isolation and/or loneliness that is delivered in‐person regardless of the intensity, duration, and frequency of administration. We will exclude digital or technology‐based interventions.

Included interventions may be one‐on‐one or group based and will be categorized based on our conceptual framework as self‐guided delivery, interpersonal delivery, community‐based delivery, societal level delivery as well as multi‐component or complex interventions. See Table 1 for subcategories and examples.

**Table 1:** Types of interventions.

Intervention categories	Subcategories	Examples
Self‐delivery	Self‐guided changing cognition	– Self‐guided mindfulness therapy – Self‐guided reminiscence therapy
	Self‐guided social skills training and psychoeducation	– Solitary social skills training – Psychoeducation, e.g., gratitude
Interpersonal delivery	Changing cognition led by a health professional	– Cognitive behavioral therapy – Mindfulness therapy – Reminiscence therapy
	Social skills training and psychoeducation led by a health professional	– Friendship enrichment program – Family therapy – Psychosocial school intervention
	Healthcare support	– Hearing aids – Social prescribing (Primary care referral to support services)
	Social support	– Community navigators
Community‐based delivery	Group activities	– Activities aimed at bringing people together through shared interests as well as facilitating social connection, e.g., education or health promotional activities (gardening, exercise, or fitness program)
	Support groups	– Group‐based interventions for people with common conditions or risk factors for social isolation or loneliness, e.g., diabetes, bereavement, caregivers
	Neighborhood approaches	– Community networks – Intergenerational approaches – Meals on wheels – Lunch clubs – Spiritual‐based programs – Built environment (changes to transportation infrastructure, housing and landscape design improvements, parks)
	Age‐friendly communities	– Dementia friendly communities – Friendly schools – World Health Organization age‐friendly communities
	Volunteering	– Volunteering
Societal level delivery	Public health, healthcare, and social policies that promote social connection, address loneliness and social isolation, facilitate social cohesion and inclusion	– Frome Model of Enhanced Primary Care
	Public education and awareness of how to develop and maintain meaningful social connection and relationships	– Campaign to end loneliness
	Policies on the urban design of neighborhoods and social infrastructure of communities	
	Policies on workplaces and how to initiate, maintain, and develop meaningful social connection with co‐workers and with the organization	– Internal employer policies and procedures that foster employee connection (group spaces, peer activities, mentoring programs), and government incentives to foster organizational change (e.g., tax credits to induce behavior change).
	Funding relevant research on implementing programs and policies and facilitating the rapid translation from evidence to practice and policy	– Funding relevant research on implementing programs and policies and to facilitate the rapid translation from evidence to practice and policy
Multi‐component or complex	‐	– Social skills training and mindfulness group counseling

Comparison interventions will include no interventions, other interventions, or usual care.

If reviews include a subset of interventions that is not eligible, we will only code studies with the eligible interventions.

#### 3.5.3 Types of population (as applicable)

We will consider any age group, people with or at risk of social isolation or loneliness, or the general population, whether based on case finding or screening for vulnerability or not.

Age groups will include:

- <10 years (children)
- 10–24 years (adolescents/youth)
- 44–60 years (middle‐aged)
- 60–75 years (youngest‐old)
- 75–85 years (middle‐old)
- >85 years (oldest‐old)

#### 3.5.4 Types of outcome measures (as applicable)

We will consider the following types of outcomes:

- health and psychosocial outcomes,
- indicators of social connections,
- cost and cost‐effectiveness outcomes, and
- process indicators (or implementation outcomes).

We will consider adverse effects of interventions such as psychological distress, safety and others as described by the studies. Different measuring tools have been used for loneliness, social isolation, and related outcomes. See Table 2 for outcome categories and measurements.

**Table 2:** Outcome categories.

Outcomes	Acceptable measurements
*Health and psychosocial outcomes*	
Loneliness	UCLA loneliness scale, de Jong‐Gierveld loneliness scale, other scales, e.g., Social and Emotional Loneliness Scale, Hughes loneliness scale
Social isolation	Lubben's Social Network Scale, Social Network Index, PROMIS social isolation 6‐I scale
Social connectedness/interactions/networks or life satisfaction	Lee and Robin's Social Connectedness Scale; Number of contacts; Frequency of social interactions; Satisfaction with interaction; Index of support satisfaction; Support network satisfaction; Companionship scale satisfaction
Well‐being/Quality of life	MOS SF‐36 Health Survey; Work and Social Adjustment Scale (WSAS); WHOQOL
Anxiety/depression	Beck Depression Inventory (BDI); Depression Adjective Check List (DACL) Form E; Geriatric depression scale; The Centre for Epidemiological Studies Depression Scale (CES‐D)
Self‐efficacy or self‐esteem	General Self‐Efficacy Scale, Rosenberg Self‐Esteem Scale
Health services use	Emergency room (ER) visits, hospitalizations, premature institutionalization
Adverse effects	Psychological distress, increases in social isolation or loneliness
*Indicators of social connections*	
Social support	Duke‐UNC Functional Social Support Questionnaire, Social support scale, social Provisions scale
Social engagement	Engagement in Meaningful Activities Survey (EMAS)
Social cohesion	The Group Cohesion Scale‐Revised; Group Therapy Experience Scale, Group Environment Questionnaire, measures of neighborhood cohesion
Social capital	The World Bank's integrated questionnaire for the measurement of social capital (SC‐IQ)
*Cost and cost‐effectiveness outcomes*	
Cost‐effectiveness	Cost‐effectiveness analysis, cost utility analysis
Healthcare or social care utilization costs	Cost of service use
Cost per participant	Cost of service use per participant
*Process indicators*	
Acceptability	Various survey tools to measure acceptability
Adherence	Various survey tools to measure adherence
Feasibility	Various survey tools to measure feasibility
Barriers	e.g., language and cultural barriers, financial accessibility, hearing or vision impairments
Increased awareness of community services	Various survey tools to measure awareness

We will not use outcomes as eligibility criteria; however, eligible studies and reviews must assess interventions with a primary objective to reduce social isolation and/or loneliness. Studies and reviews assessing interventions with a stated aim to reduce social isolation and loneliness will be included. Those that assess the effects of interventions on social isolation and/or loneliness as a primary outcome or considered other indicators of social connections, such as social support, social engagement, social cohesion, and social capital will also be included.

Studies and reviews assessing the effect of interventions on indicators of psychological wellbeing such as quality of life, anxiety or depression, with a focus on mental health rather than social isolation or loneliness will be excluded.

#### 3.5.5 Other eligibility criteria

##### Types of location/situation (as applicable)

We will include all countries. We will also classify the countries by the World Health Organization regions (African Region, Region of the Americas, South‐East Asian Region, European Region, Eastern Mediterranean Region, Western Pacific Region) (WHO, 2019) and the World Bank classification by incomes: low income economies, lower‐middle income economies, upper‐middle income economies, and high income economies (World Bank, 2022).

We will not exclude primary studies and systematic reviews that do not report the countries.

##### Types of settings (as applicable)

We will include all settings, for example, residential or personal home, nursing home or long‐term care, assisted living facilities, orphanages, schools, workplaces, community centers, and medical facilities.

### 3.6 Search methods and sources

An Information Specialist (DS) designed the search strategy which was peer‐reviewed through PRESS (Peer Review of Electronic Search Strategies) (McGowan et al., 2016). See Supporting Information: Appendix 2 for the full search strategies. We will search the following databases from inception with no date or language restrictions: Ovid MEDLINE, Embase, EBM Reviews—Cochrane Central Register of Controlled Trials, APA PsycInfo via Ovid, CINAHL via EBSCO, EBSCO (all databases except CINAHL), Global Index Medicus, ProQuest (all databases), ProQuest ERIC, Web of Science, Korean Citation Index, Russian Science Citation Index, and SciELO Citation Index via Clarivate, and Elsevier Scopus.

The reference lists of all included systematic reviews will be screened in Eppi‐Reviewer to identify additional studies.

### 3.7 Analysis and presentation

#### 3.7.1 Report structure

We will follow the reporting structure of Campbell EGMs with the standard headings: abstract, plain language summary, background, methods, results, discussion, and conclusion.

The report will include the description of the study flow with included studies, excluded studies, and any studies awaiting assessment as well as the PRISMA study flow diagram. We will also present the conceptual framework and tables and figures summarizing the distribution of included primary studies and systematic reviews across all the coding categories such as study designs, publication status, quality of systematic reviews, types of interventions, types of outcomes, population characteristics, settings, geographic distribution.

**Figure 2:**
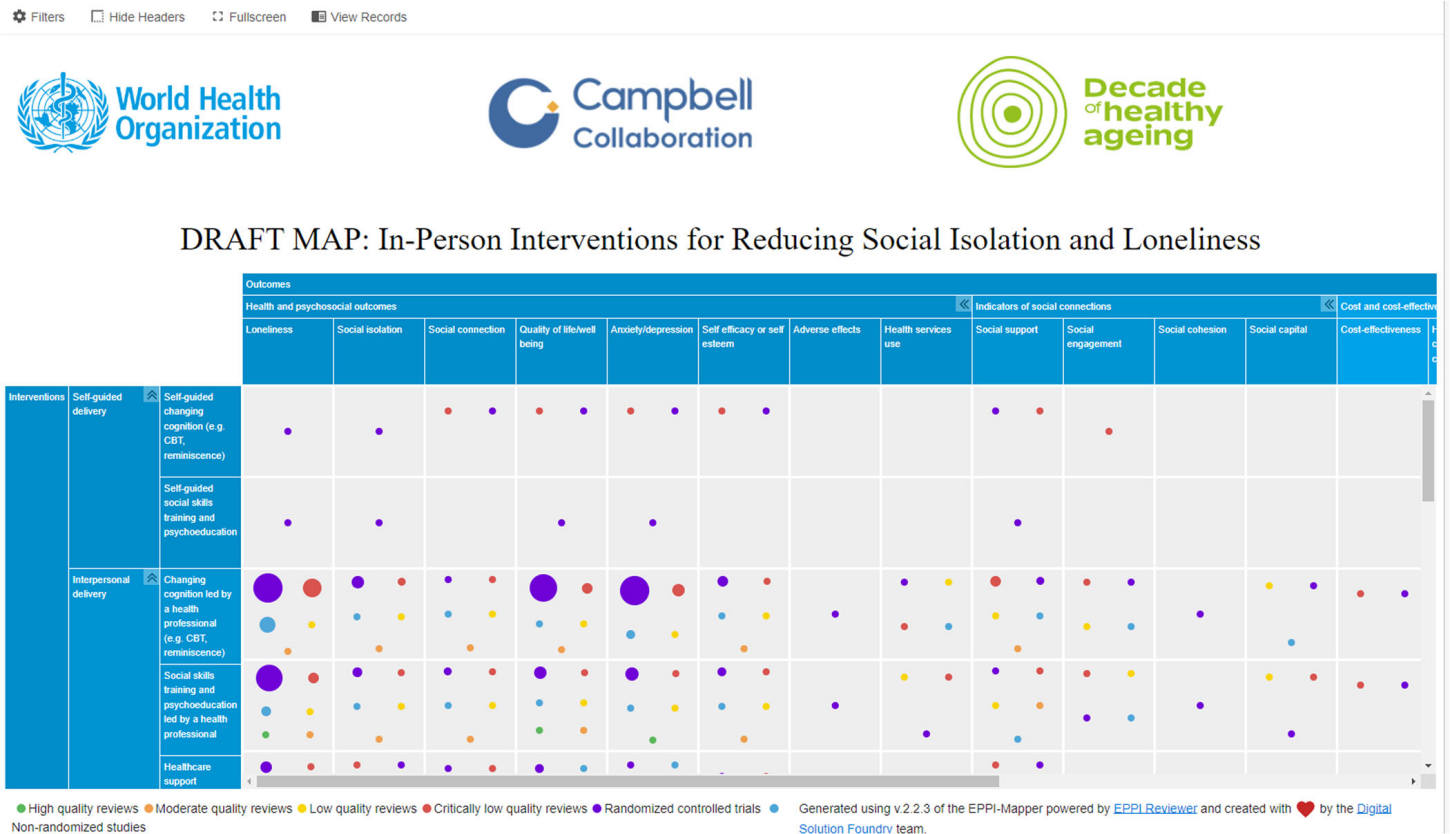
Sample map.

The evidence and gap map will have interventions as the row dimension and outcomes as the column dimension. We will use bubbles of different sizes to represent included primary studies and systematic reviews and different colors to distinguish primary studies and methodological quality of systematic reviews. The number of included studies and coded information will determine which filters will be used in the map. See a sample of the map in Figure 2.

#### 3.7.2 Filters for presentation

Additional dimensions of interest used as filters will include:

1. Study characteristics: the publication status of included studies, study design, countries where studies were conducted, World Bank classification by income (low income economies, lower‐middle income economies, upper‐middle income economies, high income economies), and WHO regions (African Region, Region of the Americas, South‐East Asian Region, European Region, Eastern Mediterranean Region, Western Pacific Region), and setting (personal home, independent living/residential home, assisted living, long‐term care/nursing home, orphanages, schools, workplaces, community centers, art gallery or museums, medical facilities, prisons), equity focus (study aimed at/focused on disadvantaged across any PROGRESS‐Plus factors) and equity analysis (assessing any differences in effects (benefit or harm) across any PROGRESS‐Plus factors).
2. Intervention characteristics: focus (loneliness, social isolation, or both); format (group‐based or one‐on‐one), sectors (clinical and population health, transport, housing, work, nutrition, environment, education, leisure: arts and entertainment, and spiritual care), goals (where changes are expected to occur—individual level, relationship level, community level, or society level), and risk factors targeted by the interventions.
3. Population characteristics: age groups, and other sociodemographic factors as well as needs.

#### 3.7.3 Equity analysis

We will assess equity following the same methods used in the evidence and gap map on digital interventions to reduce social isolation and loneliness in older adults (Welch et al., 2022). We will use the PROGRESS‐Plus acronym to describe sociodemographic inclusion factors associated with health inequities (O'Neill et al., 2014). PROGRESS‐Plus stands for Place of residence (urban/rural), Race/ethnicity/culture and language, Occupation, Gender or sex, Religion, Education, Socioeconomic status, Social capital, Plus: personal characteristics (e.g., age, disabilities), relationship features (e.g., exclusion from school, parent drug use), and time‐dependent relationships (e.g., leaving the hospital, released from prison or other times when an individual might be temporarily disadvantaged).

We will document whether studies focused on populations who are at risk or experiencing barriers to health and social care or health inequities across any PROGRESS‐Plus factors. For these studies, since interventions target different populations, we will document how potentially vulnerable populations, older people for instance, are defined and identified (e.g., using case finding, outreach, community‐based programs, screening in primary care, through formal service network or agencies).

In addition, for each study, we will assess whether studies have analyzed differential effects across PROGRESS factors (Place of residence (urban/rural), Race/ethnicity/culture and language, Occupation, Gender or sex, Religion, Education, Socioeconomic status, Social capital) for populations experiencing inequities. We will also assess analysis across additional (“Plus”) factors known to be important for special populations, including age, disability, social frailty, literacy, living status, health status.

#### 3.7.4 Dependency

We will treat multiple reports of the same study as one study. A study with multiple outcomes and interventions will be shown multiple times on the map (once for each outcome or intervention identified). Primary studies will be mapped regardless of whether they are included in multiple systematic reviews. Systematic reviews will be mapped to interventions and outcomes based on their PICO question.

### 3.8 Data collection and analysis

#### 3.8.1 Screening and study selection

Pairs of reviewers will use Machine learning text mining in Eppi‐Reviewer web‐based software program (Thomas et al., 2020) to screen titles and abstracts independently (EG, SD, EB, VB, TH, AW, AA, PD, JH, RD, SA, RI, LM, AAA, AJ, and FJ). We will initially screen 10% of the titles and abstracts. The priority screening function will develop a classifier based on the probability of inclusion determined from the preliminary screening results and present the most likely studies to be included first. We will manually screen all the articles to ensure all potentially eligible studies are captured for the full text screening stage which will also be conducted by two reviewers independently.

We will also screen the reference lists of included systematic reviews to identify additional studies that may have been missed in the database searches.

All screening will be done following the eligibility criteria (see Supporting Information: Appendix 3).

#### 3.8.2 Data extraction and management

We will develop and pilot test a data extraction code set in Eppi‐Reviewer for data collection (see draft in Supporting Information: Appendix 4). We will use a set of included studies for testing. The same studies will be coded by all the reviewers and the coding will be assessed for agreement. Any discrepancies will be discussed, and description of the coding criteria will be modified for clarity as necessary. After the pilot test, members of the team (EG, SD, EB, VB, TH, AW, AA, PD, JH, RD, SA, RI, LM, AAA, AJ, and FJ) will individually extract and code data. Non‐English papers will be coded by CWY, RY and TAMTM. Automation and text mining will not be used for coding.

We will code the study characteristics (study design, publication status, methodological quality assessment of systematic reviews), categories and subcategories of interventions and other intervention characteristics (focus, sectors, goals, and risk factors targeted), outcome domains and subdomains, population characteristics (using PROGRESS‐Plus acronym), settings, locations (by country, WHO region, and World Bank income classification).

We will code how populations were recruited and whether they were selected based on disadvantages across any PROGRESS‐Plus factors.

We will also code whether differential analysis across any PROGRESS‐Plus factors was conducted in the studies and systematic reviews to understand any equity issues.

We will not contact authors of studies or systematic reviews for any missing information given the expected size of the map (over 300 studies).

#### 3.8.3 Tools for assessing risk of bias/study quality of included reviews

Pairs of reviewers will use the AMSTAR 2 tool (Shea et al., 2017) to assess the quality of systematic reviews independently. Any disagreements will be resolved by discussion. Primary studies will not be assessed for risk of bias or methodological quality following guidance for evidence maps (Snilstveit, 2016; White et al., 2020).

#### 3.8.4 Methods for mapping

We will use the EPPI‐Mapping tool (Digital Solution Foundry and EPPI_Centre, 2020) to develop the evidence and gap map.

## CONTRIBUTIONS OF AUTHORS

The recommended optimal EGM team composition includes at least one person who has content expertise, at least one person who has methodological expertise and at least one person who has statistical expertise. It is also recommended to have one person with information retrieval expertise.

- Content: VW, EG, NH, SB, SD, JHL, ML, MLim, KM, PQ, CC, PH, CM
- EGM methods: VW, EG, PH, CM, SD, WYC, RMY, TAMTM
- Information retrieval: DS

## DECLARATIONS OF INTEREST

Vivian Welch is editor in chief of the Campbell Collaboration. The editorial process was handled by an independent editor and VW had no input in the editorial process or decisions.

Julianne Holt‐Lunstad and Michelle Lim were co‐investigators on the KIND challenge intervention aimed at reducing loneliness which was sponsored by the app Nextdoor.

Dr. Kate Mulligan has been a contract consultant for the Canadian Red Cross, a provider of in‐person interventions to reduce social isolation and loneliness. She has also been involved in intervention projects related to social prescribing with the Alliance for Healthier Communities.

Pamela Qualter was involved in two reviews used to gather data for the current evidence gap review; she was not involved in any primary research in the subject area of the review.

Sierra Dowling is the managing editor of the Campbell Ageing Coordinating Group, but the editorial process was handled by another managing editor.

Paul Hebert is the Chief Medical and Science advisor for the Canadian Red Cross.

Christopher Mikton works for the World Health Organization which has helped fund this evidence and gap map.

Elizabeth Ghogomu, Wan Yuen Choo, Raudah Mohd Yunus, Tengku Amatullah Madeehah Tengku Mohd, Niobe Haitas, Sivan Bomze, Simone Dahrouge, Edward Garcia, Mathias Lasgaard, and Douglas M. Salzwedel, have no conflicts of interest.

## PLANS FOR UPDATING EGM

The EGM will be updated every 2 years.

## SOURCES OF SUPPORT

1

Internal sources

- None, Other

2

External sources

- World Health Organization, Switzerland

Purchase Order Number: 202759417

## Supporting information

Supporting information.
