# Supplementary material for: PROTOCOL: In‐person interventions to reduce social isolation and loneliness: An evidence and gap map
Source: Campbell Syst Rev. 2023 Jun 22;19(3):e1340. doi: 10.1002/cl2.1340 (PMC10286723; doi:10.1002/cl2.1340)
Supplement: Supplementary file 1 — Supporting information. [file CL2-19-e1340-s001.docx]

Appendices

1 Glossary of terms

**Social capital**: refers to a series of resources that individuals earn as a result of their membership in social networks, and the features of those networks that facilitate individual or collective actions such as interpersonal trust, reciprocity and mutual aid (Bethell et al., 2019; Elder et al., 2012; Mann et al., 2017; Zavaleta et al., 2014).

**Social cohesion**: is the neighborhood-based resource of mutual community trust and solidarity (Elder et al., 2012).

**Social connection**: encompasses the structural, functional, and quality aspects of how individuals connect to each other (Badcock et al., 2022; Holt-Lunstad 2018; NASEM 2020). It means having a variety of relationships (from close personal ties such as family and friends through to weaker ties such as acquaintances and strangers); relationships you can rely upon for support; and relationships that are trusted, high quality, and satisfying. These relationships reflect a multitude of influences, including the diversity of our individual cultures and experiences and one’s biology (Badcock et al., 2022).

**Social connectedness**: the degree to which an individual or population falls along the continuum of social connection (Badcock et al., 2022).

**Social engagement**: reflects participation in meaningful activities with others (Berkman et al., 2000; Bethell et al., 2019; Elder et al., 2012).

**Social network**: denotes the number and type of social relationships with people (individuals or groups) (Berkman et al., 2000; Bethell et al., 2019; Kelly et al., 2017; Mann et al., 2017).

**Social support**: the actual or perceived availability of resources (e.g., informational, tangible, emotional help) from others, typically one’s social network (Berkman et al., 2000; Bethell et al., 2019; Kelly et al., 2017; Mann et al., 2017) to help them adapt to change and cope with stress (Elder et al., 2012).

## 2 Search strategies

Database: Ovid MEDLINE(R) ALL <1946 to February 16, 2022>

Search Date: 17 February 2022

--------------------------------------------------------------------------------

1 loneliness.mp. (10313)

2 (loneli* or lonely).tw,kf. (10332)

3 *social isolation/ (6701)

4 (social exclus* or social* isolat*).tw,kf. (13293)

5 or/1-4 (26870)

6 *social support/ (28664)

7 (social capital or social cohesion or social contact? or social connect* or social integration or social* interact* or social network? or social participat* or social relation* or social skill*).ti,kf. (19799)

8 (social capital or social cohesion or social contact? or social connect* or social integration or social* interact* or social network? or social participat* or social relation* or social skill*).ab. /freq=2 (21335)

9 ((peer or psychological or psychosocial) adj (counsel* or support*)).tw,kf. (17038)

10 social prescribing.tw,kf. (242)

11 *counseling/ (17136)

12 mindfulness/ or mindfulness-based interventions/ (4980)

13 (meditat* or mindfulness).tw,kf. (15237)

14 psychotherapy/mt, st (14296)

15 (psychoeducation* or psychotherap*).tw,kf. (55754)

16 stress, psychological/th (6064)

17 ((improv* or measure*) adj6 (wellbeing or well-being)).tw,kf. (19221)

18 ((animal or dog or dogs or pet or pets) adj2 therap*).tw,kf. (3131)

19 or/6-18 (187036)

20 ((benefit* or change or changes or contribut* or decreas* or develop* or effect or effects or effectiveness or enhance* or evaluat* or experience* or experiment* or impact* or implement* or improv* or increas* or intervention* or method* or outcome* or pilot* or program* or provid* or reduc* or study or support* or system* or target* or technolog* or training or trial or “use of “) adj3 (loneliness or lonely or social isolat*)).ti,kf. (977)

21 ((benefit* or change or changes or contribut* or decreas* or develop* or effect or effects or effectiveness or enhance* or evaluat* or experience* or experiment* or impact* or implement* or improv* or increas* or intervention* or method* or outcome* or pilot* or program* or provid* or reduc* or study or support* or system* or target* or technolog* or training or trial or “use of “) adj3 (loneliness or lonely or social isolat*)).ab. (5290)

22 or/20-21 (5631)

23 systematic review.mp,pt. (246797)

24 meta analysis.mp,pt. (236570)

25 (cochrane or embase or medline or pubmed).ab. (278100)

26 randomized controlled trial.pt. (558823)

27 controlled clinical trial.pt. (94699)

28 pragmatic clinical trial.pt. (2033)

29 randomi*.tw,kf. (731378)

30 placebo.ab. (225725)

31 clinical trials as topic/ (199206)

32 (randomly adj2 (allocated or assigned)).ab. (157178)

33 trial.ti. (256921)

34 (group or groups).ab. /freq=2 (2060696)

35 ((quasi experiment* or quasiexperiment* or quasi randomi* or quasirandomi*) adj2 (design* or method* or study or trial)).ab,kf. (12777)

36 ((before adj5 after) or (controlled adj3 study) or (controlled adj3 trial) or control group* or effect* or evaluat* or (pre adj5 post) or ((pretest or pre-test) and (posttest or post test))).tw. (10522457)

37 controlled before-after studies/ (680)

38 interrupted time series analysis/ (1526)

39 (time series adj5 (analys* or design* or interrupted or ITS or studies or study or trial)).ab,kf. (15364)

40 or/23-39 (11690740)

41 animals/ not (humans/ and animals/) (4926316)

42 40 not 41 (9499796)

43 5 and (19 or 22) and 42 (4412)

--------------------------------------------------------------------------------

Database: Embase Classic+Embase <1947 to 2022 February 16>

Search Date: 17 February 2022

--------------------------------------------------------------------------------

1 *loneliness/ (3846)

2 (loneli* or lonely).tw,kf. (12763)

3 *social isolation/ (7441)

4 (social exclus* or social* isolat*).tw,kf. (17528)

5 or/1-4 (32997)

6 *social support/ (24839)

7 (social capital or social cohesion or social contact? or social connect* or social integration or social* interact* or social network? or social participat* or social relation* or social skill*).tw,kf. (90221)

8 ((peer or psychological or psychosocial) adj (counsel* or support*)).tw,kf. (25690)

9 *counseling/ (18490)

10 *mindfulness/ (5758)

11 (meditat* or mindfulness).tw,kf. (20399)

12 exp *psychotherapy/ (141787)

13 (psychoeducation* or psychotherap*).tw,kf. (81789)

14 exp *mental stress/ (70077)

15 ((improv* or measure*) adj6 (wellbeing or well-being)).tw,kf. (24927)

16 ((animal or dog or dogs or pet or pets) adj2 therap*).tw,kf. (4910)

17 or/6-16 (431837)

18 ((benefit* or change or changes or contribut* or decreas* or develop* or effect or effects or effectiveness or enhance* or evaluat* or experience* or experiment* or impact* or implement* or improv* or increas* or intervention* or method* or outcome* or pilot* or program* or provid* or reduc* or study or support* or system* or target* or technolog* or training or trial or “use of “) adj3 (loneliness or lonely or social isolat*)).ti,kf. (1083)

19 ((benefit* or change or changes or contribut* or decreas* or develop* or effect or effects or effectiveness or enhance* or evaluat* or experience* or experiment* or impact* or implement* or improv* or increas* or intervention* or method* or outcome* or pilot* or program* or provid* or reduc* or study or support* or system* or target* or technolog* or training or trial or “use of “) adj3 (loneliness or lonely or social isolat*)).ab. (6634)

20 or/18-19 (7011)

21 systematic review.tw. (260439)

22 meta analysis.tw. (248362)

23 (cochrane or embase or medline or pubmed).ab. (351613)

24 randomized controlled trial/ (698639)

25 crossover procedure/ (69804)

26 double-blind procedure/ (194902)

27 (randomi?ed or randomly).tw. (1415368)

28 (crossover* or cross-over*).tw. (118001)

29 placebo.ab. (332194)

30 (doubl* adj blind*).tw. (233178)

31 assign*.ab. (440230)

32 allocat*.ab. (175384)

33 ((quasi experiment* or quasiexperiment* or quasi randomi* or quasirandomi*) adj2 (design* or method* or study or trial)).ab,kf. (15630)

34 ((before adj5 after) or (controlled adj3 study) or (controlled adj3 trial) or control group* or effect? or evaluat* or intervention* or (pre adj5 post) or ((pretest or pre-test) and (posttest or post test))).ab,kf. /freq=2 (5797988)

35 time series analysis/ (31515)

36 (time series adj5 (analys* or design* or interrupted or ITS or studies or study or trial)).ab,kf. (18097)

37 or/21-36 (7230810)

38 (exp animal/ or animal.hw. or nonhuman/) not (exp human/ or human cell/ or (human or humans).ti.) (7569320)

39 37 not 38 (5733318)

40 5 and (17 or 20) and 39 (3924)

41 limit 40 to embase (1799)

--------------------------------------------------------------------------------

Database: EBM Reviews - Cochrane Central Register of Controlled Trials <January 2022>

Search Date: 17 February 2022

--------------------------------------------------------------------------------

1 (loneli* or lonely).mp. (957)

2 (social exclus* or social* isolat*).mp. (1243)

3 or/1-2 (2044)

4 (social capital or social cohesion or social contact? or social connect* or social integration or social* interact* or social network? or social participat* or social relation* or social skill*).mp. (9267)

5 ((peer or psychological or psychosocial) adj (counsel* or support*)).mp. (3325)

6 social prescribing.mp. (16)

7 (counseling or counselling).tw. (21179)

8 (meditat* or mindfulness).mp. (8535)

9 psychoeducation*.mp. (4415)

10 ((improv* or measure*) adj6 (wellbeing or well-being)).tw. (27292)

11 ((animal or dog or dogs or pet or pets) adj2 therap*).tw. (459)

12 or/4-11 (69609)

13 ((benefit* or change or changes or contribut* or decreas* or develop* or effect or effects or effectiveness or enhance* or evaluat* or experience* or experiment* or impact* or implement* or improv* or increas* or intervention* or method* or outcome* or pilot* or program* or provid* or reduc* or study or support* or system* or target* or technolog* or training or trial or “use of “) adj3 (loneliness or lonely or social isolat*)).tw. (710)

14 3 and (12 or 13) (1114)

--------------------------------------------------------------------------------

Database: APA PsycInfo <1806 to February Week 1 2022>

Search Date: 17 February 2022

--------------------------------------------------------------------------------

1 loneliness.mp. (13276)

2 (loneli* or lonely).tw. (12931)

3 *social isolation/ (6142)

4 (social exclus* or social* isolat*).tw. (13479)

5 or/1-4 (29499)

6 *social support/ (29322)

7 (social capital or social cohesion or social contact? or social connect* or social integration or social* interact* or social network? or social participat* or social relation* or social skill*).ti. (23403)

8 (social capital or social cohesion or social contact? or social connect* or social integration or social* interact* or social network? or social participat* or social relation* or social skill*).ab. /freq=2 (31997)

9 ((peer or psychological or psychosocial) adj (counsel* or support*)).tw. (12057)

10 social prescribing.tw. (79)

11 *counseling/ (20204)

12 mindfulness/ or mindfulness-based interventions/ (12465)

13 (meditat* or mindfulness).tw. (23510)

14 (psychoeducation* or psychotherap*).tw. (137178)

15 ((improv* or measure*) adj6 (wellbeing or well-being)).tw. (14941)

16 ((animal or dog or dogs or pet or pets) adj2 therap*).tw. (1127)

17 or/6-16 (266029)

18 ((benefit* or change or changes or contribut* or decreas* or develop* or effect or effects or effectiveness or enhance* or evaluat* or experience* or experiment* or impact* or implement* or improv* or increas* or intervention* or method* or outcome* or pilot* or program* or provid* or reduc* or study or support* or system* or target* or technolog* or training or trial or “use of “) adj3 (loneliness or lonely or social isolat*)).ti. (946)

19 ((benefit* or change or changes or contribut* or decreas* or develop* or effect or effects or effectiveness or enhance* or evaluat* or experience* or experiment* or impact* or implement* or improv* or increas* or intervention* or method* or outcome* or pilot* or program* or provid* or reduc* or study or support* or system* or target* or technolog* or training or trial or “use of “) adj3 (loneliness or lonely or social isolat*)).ab. (5335)

20 or/18-19 (5699)

21 systematic review.tw. (36030)

22 meta analys*.tw. (41839)

23 (cochrane or embase or medline or pubmed).ab. (30072)

24 randomi*.tw. (98659)

25 placebo.ab. (41519)

26 (randomly adj2 (allocated or assigned)).ab. (44497)

27 trial.ti. (34469)

28 (group or groups).ab. /freq=2 (430004)

29 ((quasi experiment* or quasiexperiment* or quasi randomi* or quasirandomi*) adj2 (design* or method* or study or trial)).ab. (9473)

30 ((before adj5 after) or (controlled adj3 study) or (controlled adj3 trial) or control group* or evaluat* or (pre adj5 post) or ((pretest or pre-test) and (posttest or post test))).ab. (690568)

31 or/21-30 (1101796)

32 5 and (17 or 20) and 31 (2485)

--------------------------------------------------------------------------------

Database: CINAHL via EBSCO

Search Date: 17 February 2022

Yield: 1308

--------------------------------------------------------------------------------

S39 S38 Limiters - Exclude MEDLINE records

1,308

S38 S4 AND (S17 OR S18) AND S37 2,531

S37 S36 NOT S35 1,440,435

S36 (S19 OR S20 OR S21 OR S22 OR S23 OR S24 OR S25 OR S26 OR S27 OR S28 OR S29 OR S30) 1,498,022

S35 S33 NOT S34 120,393

S34 MH human 2,503,617

S33 (S32 OR S33) 144,244

S32 MH animal studies 144,237

S31 MH Animals+ 99,119

S30 AB (time series N5 (analys* OR design* OR interrupted OR ITS OR studies OR study OR trial)) 4,647

S29 AB (before N5 after) OR (controlled N3 study) OR (controlled N3 trial) OR control group* OR (pre N5 post) OR ((pretest OR pre-test) and (posttest OR post test)) 486,011

S28 TX (quasi experiment* OR quasiexperiment* OR quasi randomi* OR quasirandomi*) N2 (design* OR method* OR study OR trial) 21,095

S27 MH (“Controlled Before-After Studies” OR “Interrupted Time Series Analysis”) 942

S26 MH (crossover design OR comparative studies) 441,548

S25 TI ( ((singl* N1 blind*) OR (doubl* N1 blind*))) OR AB ( ((singl* N1 blind*) OR (doubl* N1 blind*))) 53,623

S24 TI (randomi* OR trial) OR ( AB (allocat* OR assign* OR control* OR randomi* OR randomly)) 932,731

S23 (MH “Randomized Controlled Trials”) OR (MH “Single-Blind Studies”)OR (MH “Double-Blind Studies”) OR (MH “Triple-Blind Studies”) OR (MH “Random Assignment”) OR MH (“Intervention Trials”) OR MH (“Crossover Design”) OR MH (“Cluster Sample”) 213,707

S22 PT randomized controlled trial 139,239

S21 AB (cochrane OR embase OR medline OR pubmed OR searched) 123,048

S20 TI ( (systematic review) OR TI meta analy* OR TI metaanaly*) OR AB ( (systematic review) OR TI meta analy* OR TI metaanaly*) 153,494

S19 (MH “Systematic Review”) OR (MH “Meta Analysis”) 135,353

S18 ( TI (benefit* OR change OR changes OR contribut* OR decreas* OR develop* OR effect OR effects OR effectiveness OR enhance* OR evaluat* OR experience* OR experiment* OR impact* OR implement* OR improv* OR increas* OR intervention* OR method* OR outcome* OR pilot* OR program* OR provid* OR reduc* OR study OR support* OR system* OR target* OR technolog* OR training OR trial OR “use of “) N3 (loneliness OR lonely OR social isolat*)) OR ( AB (benefit* OR change OR changes OR contribut* OR decreas* OR develop* OR effect OR effects OR effectiveness OR enhance* OR evaluat* OR experience* OR experiment* OR impact* OR implement* OR improv* OR increas* OR intervention* OR method* OR outcome* OR pilot* OR program* OR provid* OR reduc* OR study OR support* OR system* OR target* OR technolog* OR training OR trial OR “use of “) N3 (loneliness OR lonely OR social isolat*)) 4,381

S17 S5 OR S6 OR S7 OR S8 OR S9 OR S10 OR S11 OR S12 OR S13 OR S14 OR S15 OR S16 278,300

S16 TX (animal OR dog OR dogs OR pet OR pets) N2 therap*) 2,852

S15 TX (improv* OR measure*) N6 (wellbeing OR well-being) 12,195

S14 (MH “Stress, Psychological”) 55,437

S13 TI (psychotherap* OR psychoeducation*) OR AB (psychotherap* OR psychoeducation*) 21,037

S12 (MH “Psychotherapy”) 22,713

S11 TX (meditat* OR mindfulness) 15,996

S10 (MH “Mindfulness”) 6,551

S9 (MH “Counseling”) 32,328

S8 TI social prescribing OR AB social prescribing 297

S7 ( TI (peer OR psychological OR psychosocial) N1 (counsel* OR support*)) OR ( AB (peer OR psychological OR psychosocial) N1 (counsel* OR support*)) 12,904

S6 ( TI (social capital OR social cohesion OR social contact? OR social connect* OR social integration OR social* interact* OR social network? OR social participat* OR social relation* OR social skill*)) OR ( AB (social capital OR social cohesion OR social contact? OR social connect* OR social integration OR social* interact* OR social network? OR social participat* OR social relation* OR social skill*)) 60,583

S5 (MH “Support, Psychosocial”) 89,639

S4 (S1 OR S2 OR S3) 21,689

S3 ( TI (social isolation OR social exclusion)) OR ( AB (social isolation OR social exclusion)) 7,219

S2 ( TI (loneli* OR lonely)) OR ( AB (loneli* OR lonely)) 6,889

S1 (MH “Social Isolation+“) 14,762

--------------------------------------------------------------------------------

Database: EBSCO (all databases except CINAHL)

Search Date: 19 February 2022

--------------------------------------------------------------------------------

S5 S1 AND S2 AND S3 AND S4 1,124 (883 after automatic removal of duplicates upon export)

S4 TX (“controlled before-after” OR “before-after trial” OR “controlled trial” OR “double-blind*“ OR “meta-analysis” OR “quasi-experiment*“ OR quasiexperiment* OR randomi* OR randomly OR “single-blind*“ OR “systematic review” OR “time series”) 4,030,933

S3 TX (benefit* OR change OR changes OR contribut* OR decreas* OR develop* OR effect OR effects OR effectiveness OR enhance* OR evaluat* OR experience* OR experiment* OR impact* OR implement* OR improv* OR increas* OR intervention* OR method* OR outcome* OR pilot* OR program* OR provid* OR reduc* OR study OR support* OR system* OR target* OR technolog* OR training OR trial OR “use of “) 31,183,298

S2 TX (“animal therap*“ OR befriend* OR connected* OR counsel* OR meditat* OR mindfulness* OR “pet therap*“ OR psychoeducat* OR psychologic* OR psychosocial* OR psychotherap* OR “social capital” OR “social cohesion” OR “social contact” OR “social connect*“ OR “social integration” OR “social interact*“ OR “social network*“ OR “social participat*“ OR “social prescribing” OR “social relation*“ OR “social skill*“ OR “social support*“ OR “well-being” OR wellbeing) 420,808

S1 TI (loneli* OR lonely OR “social exclusion” OR “social isolation” OR “socially isolated”) 32,902

--------------------------------------------------------------------------------

Database: Global Index Medicus

Search Date: 19 February 2022

Yield: 80

--------------------------------------------------------------------------------

tw:((tw:((loneli* OR lonely OR “social exclusion” OR “social isolation” OR “socially isolated”))) AND (tw:((“animal therapy” OR befriend* OR connected* OR counsel* OR meditat* OR mindfulness* OR “pet therapy” OR psychoeducat* OR psychologic* OR psychosocial* OR psychotherap* OR “social capital” OR “social cohesion” OR “social contact” OR “social connection” OR “social integration” OR “social interaction” OR “social network” OR “social participation” OR “social prescribing” OR “social relations” OR “social skills” OR “social support” OR “well-being” OR wellbeing OR benefit* OR change OR changes OR contribut* OR decreas* OR develop* OR effect OR effects OR effectiveness OR enhance* OR evaluat* OR experience* OR experiment* OR impact* OR implement* OR improv* OR increas* OR intervention* OR method* OR outcome* OR pilot* OR program* OR provid* OR reduc* OR study OR support* OR system* OR target* OR technolog* OR training OR trial OR “use of “)))) AND ( type_of_study:(“systematic_reviews” OR “evaluation_studies” OR “clinical_trials”))

--------------------------------------------------------------------------------

Database: ProQuest (all databases)

Search Date: 19 February 2022

Yield: 539

--------------------------------------------------------------------------------

ti((loneli* OR lonely OR “social exclusion” OR “social isolation” OR “socially isolated”)) AND noft((“animal therapy” OR befriend* OR connected* OR counsel* OR meditat* OR mindfulness* OR “pet therapy” OR psychoeducat* OR psychologic* OR psychosocial* OR psychotherap* OR “social capital” OR “social cohesion” OR “social contact” OR “social connection” OR “social integration” OR “social interaction” OR “social network” OR “social participation” OR “social prescribing” OR “social relations” OR “social skills” OR “social support” OR “well-being” OR wellbeing)) AND noft((benefit* OR change OR changes OR contribut* OR decreas* OR develop* OR effect OR effects OR effectiveness OR enhance* OR evaluat* OR experience* OR experiment* OR impact* OR implement* OR improv* OR increas* OR intervention* OR method* OR outcome* OR pilot* OR program* OR provid* OR reduc* OR study OR support* OR system* OR target* OR technolog* OR training OR trial OR “use of “)) AND noft((“controlled before-after” OR “before-after trial” OR “controlled trial” OR “double-blind” OR “meta-analysis” OR “quasi-experimental” OR quasiexperiment* OR randomi* OR randomly OR “single-blind” OR “systematic review” OR “time series”))

--------------------------------------------------------------------------------

Database: ProQuest ERIC

Search Date: 20 February 2022

Yield: 104

--------------------------------------------------------------------------------

(loneli* OR lonely OR “social exclusion” OR “social isolation” OR “socially isolated”) AND (“animal therapy” OR befriend* OR connected* OR counsel* OR meditat* OR mindfulness* OR “pet therapy” OR psychoeducat* OR psychologic* OR psychosocial* OR psychotherap* OR “social capital” OR “social cohesion” OR “social contact” OR “social connection” OR “social integration” OR “social interaction” OR “social network” OR “social participation” OR “social prescribing” OR “social relations” OR “social skills” OR “social support” OR “well-being” OR wellbeing OR benefit* OR change OR changes OR contribut* OR decreas* OR develop* OR effect OR effects OR effectiveness OR enhance* OR evaluat* OR experience* OR experiment* OR impact* OR implement* OR improv* OR increas* OR intervention* OR method* OR outcome* OR pilot* OR program* OR provid* OR reduc* OR study OR support* OR system* OR target* OR technolog* OR training OR trial OR “use of “) AND (“controlled before-after” OR “before-after trial” OR “controlled trial” OR “double-blind” OR “meta-analysis” OR “quasi-experimental” OR quasiexperiment* OR randomi* OR randomly OR “single-blind” OR “systematic review” OR “time series”)

--------------------------------------------------------------------------------

Database: Clarivate (Web of Science, KCI-Korean Citation Index, Russian Science Citation Index, SciELO Citation Index)

Search Date: 20 February 2022

Yield: 31 SciELO, 45 KCI, 549 WoS

--------------------------------------------------------------------------------

Topic = (loneli* OR lonely OR “social exclusion” OR “social isolation” OR “socially isolated”) AND (“animal therapy” OR befriend* OR connected* OR counsel* OR meditat* OR mindfulness* OR “pet therapy” OR psychoeducat* OR psychologic* OR psychosocial* OR psychotherap* OR “social capital” OR “social cohesion” OR “social contact” OR “social connection” OR “social integration” OR “social interaction” OR “social network” OR “social participation” OR “social prescribing” OR “social relations” OR “social skills” OR “social support” OR “well-being” OR wellbeing OR benefit* OR change OR changes OR contribut* OR decreas* OR develop* OR effect OR effects OR effectiveness OR enhance* OR evaluat* OR experience* OR experiment* OR impact* OR implement* OR improv* OR increas* OR intervention* OR method* OR outcome* OR pilot* OR program* OR provid* OR reduc* OR study OR support* OR system* OR target* OR technolog* OR training OR trial OR “use of “) AND (“controlled before-after” OR “before-after trial” OR “controlled trial” OR “double-blind” OR “meta-analysis” OR “quasi-experimental” OR quasiexperiment* OR randomi* OR randomly OR “single-blind” OR “systematic review” OR “time series”)

--------------------------------------------------------------------------------

Database: Elsevier Scopus

Search Date: 20 February 2022

Yield: 1834

--------------------------------------------------------------------------------

( TITLE ( ( loneli* OR lonely OR “social exclusion” OR “social isolation” OR “socially isolated”)) OR KEY ( ( loneli* OR lonely OR “social exclusion” OR “social isolation” OR “socially isolated”)) AND TITLE-ABS-KEY ( ( “animal therapy” OR befriend* OR connected* OR counsel* OR meditat* OR mindfulness* OR “pet therapy” OR psychoeducat* OR psychologic* OR psychosocial* OR psychotherap* OR “social capital” OR “social cohesion” OR “social contact” OR “social connection” OR “social integration” OR “social interaction” OR “social network” OR “social participation” OR “social prescribing” OR “social relations” OR “social skills” OR “social support” OR “well-being” OR wellbeing)) AND TITLE-ABS-KEY ( ( benefit* OR change OR changes OR contribut* OR decreas* OR develop* OR effect OR effects OR effectiveness OR enhance* OR evaluat* OR experience* OR experiment* OR impact* OR implement* OR improv* OR increas* OR intervention* OR method* OR outcome* OR pilot* OR program* OR provid* OR reduc* OR study OR support* OR system* OR target* OR technolog* OR training OR trial OR “use of “)) AND TITLE-ABS-KEY ( ( “controlled before-after” OR “before-after trial” OR “controlled trial” OR “double-blind” OR “meta-analysis” OR “quasi-experimental” OR quasiexperiment* OR randomi* OR randomly OR “single-blind” OR “systematic review” OR “time series”)))

## 3 Detailed eligibility criteria

| **Criteria** | **Inclusion** | **Exclusion** |
| --- | --- | --- |
| Population | Any age groups | Hospitalized patients |
| Intervention | In-person interventions for SIL  - **Self delivery interventions**   - Changing cognition (e.g., self-guided mindfulness, reminiscence therapy) - Social skills training and psychoeducation   **- Interpersonal delivery interventions**   - Changing cognition (e.g., cognitive behavioral therapy, reminiscence therapy) - Social skills training and psychoeducation - Healthcare support (e.g., assistive devices like hearing aids, cochlear implants; referral from primary care to social services) - Social support (e.g., social support from family and friends, befriending programs)   - **Community-based delivery interventions**   - Group activities (gardening, physical exercise) - Support groups (care givers of people with dementia, cancer patients) - Neighborhood approaches (networks, transportation, meals on wheels, or intergenerational approaches) - Age friendly communities (e.g., Dementia-friendly communities, friendly schools) - Volunteering   - **Societal level delivery interventions**   - Public health policies that promote social connection, address loneliness and social isolation, facilitate social cohesion and inclusion (e.g., policies against discrimination and marginalization (ageism, racism), socio-economic inequality, digital divide, intergenerational solidarity, social norms) - Public education and awareness on social relationships - Policies on design of neighborhoods and community social infrastructures - Funding relevant research   - **Multi-component or complex interventions** | - Remotely delivered or digital interventions (e.g. online cognitive behavioral therapy, video chat rooms, social networking sites, telephone befriending, robots, computer and internet training) |
| Evidence/aim | Focus on improving SIL, mental health and well-being | Not focused on SIL (e.g., CBT for dementia) **even if SIL measured as outcome** |
| Study design | - Systematic reviews  -*Primary studies with a comparison/control group  - Randomized controlled trials  - Quasi-experimental studies  - Controlled before-after studies  - Interrupted time series with at least six data points (3 before and 3 after a discrete intervention)  - Regression discontinuity designs  - Protocols of systematic reviews and eligible primary studies | - Not a systematic review (e.g., literature reviews) – does not satisfy at least 4/5 criteria  (i) Were inclusion/exclusion criteria reported?  (ii) Was the search adequate? (iii) Were the included studies synthesized?  (iv) Was the quality of the included studies assessed?  (v) Are sufficient details about the individual included studies presented?  - **Scoping reviews if not about effectiveness**  - less than six period interrupted time series design  - longitudinal cohort studies with no controls  - Cross-sectional studies  - Qualitative study designs  - Association studies |

## 4 Coding tool

| **Categories** | **Sub-categories (if applicable)** |
| --- | --- |
| Publication status | - Complete - On-going (e.g., protocols, trial registrations) - Conference abstract |
| Study design | - Systematic review - Primary study |
| Primary study design | - Randomized controlled trials (RCTs) - Non-randomized studies |
| Equity focus: Is the study population identified by the authors as aimed at/focused on disadvantaged across any PROGRESS-Plus factors | - Place of residence (rural or remote/urban) - Race/Ethnicity/culture/language - Occupation - Gender or Sex - Religion - Education - Socioeconomic status - Social capital (e.g., marital status – widowed, divorced, separated) - Plus-factor – social isolated or at risk - Plus-factor – lonely or at risk - Plus-factor – age (older adults, children) - Plus-factor – disability - Plus-factor – frailty - Plus-factor – health status (e.g., dementia, autism, disease severity) - Plus-factor – living situation (e.g., alone, long term care, away from home) |
| If population is identified as “at risk”, how are they identified? | - Case-finding (methods for detecting people who are at risk for a particular condition e.g., assessment using a questionnaire or scale) - Outreach (e.g., making contact with organizations, groups, specific audiences or the general public to identify people who are at risk) - From a community-based program (people at risk who participate in a community program e.g. meals on wheels, dance classes, exercise programs, book clubs, community garden, community network, etc.) - Screening in primary care - Through formal service network or agencies (e.g., Army, Veterans affairs, penitentiary/prisons, employment agencies, institutional networks, schools) |
| Quality assessment of reviews (using AMSTAR 2) | - Critically low-quality reviews - Low quality reviews - Moderate quality reviews - High quality reviews - Randomized control trials - Non-randomized studies |
| Interventions | |
| Self delivery | - Self-guided changing cognition (e.g., self-guided mindfulness) - Self-guided social skills training and psychoeducation |
| Interpersonal delivery | - Changing cognition led by ahealth professional (e.g., cognitive behavioural therapy, reminiscence therapy, mindfulness therapy) - Social skills training and psychoeducation led by a health professional - Healthcare support (e.g., referral from primary care to social services, hearing aids) - Social support (social support by family and friends, befriending programs, pet/animal-assisted therapy) |
| Community-based delivery | - Group activities (group activities aimed at other purposes e.g., education, gardening, arts, health promotion activities (exercise) - Support groups (group-based interventions for people with common illness or condition or who share common underlying causes of SIL [e.g., bereavement] - Neighborhood approaches (e.g., networks, transportation, built environment, intergenerational approaches, meals on wheels, lunch club, spiritual-based programs, other support care services) - Age-friendly communities (e.g., Dementia-Friendly Communities, friendly schools) - Volunteering |
| Societal approaches | - Public health policy that promotes social cohesion and inclusion - Public education and awareness on social relationships - Policies on design of neighbourhoods and community social infrastructures - Funding relevant research |
| Multi-component/complex | Combination of multiple interventions involving same/different levels in the same study |
| Intervention focus | - Loneliness - Social isolation - Social isolation and loneliness |
| Intervention format | - Group-based - One-on-one |
| Intervention sector | - Clinical and population health - Transport - Housing - Work - Nutrition - Environment - Education - Leisure: arts, and entertainment - Spiritual care |
| Intervention goals (where changes are expected to occur) | - Individual level - Relationship level - Community level - Society level |
| Risk factors targeted by interventions | - Specified - Unspecified |
| Outcomes | |
| Outcomes | - Health and psychosocial outcomes   - Loneliness   - Social isolation   - Social connection (social interaction, satisfaction with interactions or support network satisfaction, or companionship satisfaction, frequency of social interactions, number of contacts)   - Quality of life/well being   - Anxiety/depression   - Self-efficacy or self esteem   - Adverse effects (e.g., psychological distress, increase in social isolation or loneliness)   - Health services use (e.g., ER visits, hospitalizations, premature institutionalization) - Indicators of social connnection   - Social support   - Social engagement or participation   - Social cohesion   - Social capital - Cost and cost-effectiveness outcomes   - Cost-effectiveness   - Healthcare or social care utilization costs   - Cost per participant - Process indicators   - Acceptance   - Adherence   - Feasibility   - Increased awareness of community services   - barriers |
| Population sociodemographic | |
| Age groups | - Includes <10 years (children) - Includes 10-24 years (adolescents/youth) - Includes 25-44 years (young adults) - Includes 44-60 years (middle-aged) - Includes 60-75 years (youngest-old) - Includes 75-85 years (middle-old) - Includes >85 years (oldest-old) - Unspecified |
| Health condition | - Communicable disease (e.g., respiratory infections, pneumonia) - Non-communicable disease (e.g., diabetes, hypertension, COPD) - Autism, Asperger syndrome, ADHD - Dementia - Mental health disorders - Depression - Comorbidity (multiple health conditions) - Disability - Physical frailty (decline in nutrition, mobility, physical activity, strength, endurance, balance, sensory functions) - Psychological frailty (decline in cognition, mood, and coping) - Social frailty (decline in social relations and social support) - Care dependent - Discharge from hospital - End of life/palliative care |
| Place of residence | - Urban - Rural/remote - Unspecified |
| Race/Ethnicity/Language/Culture | - Tick if reported |
| Occupation | - Tick if reported |
| Gender or Sex | - Men/boys only - Women/girls only - LGBTQIA2S+ - Unspecified |
| Religion | - Tick if reported |
| Education | - Tick if reported |
| Socioeconomic status | - Tick if reported |
| Marital status | - Tick if reported |
| Living alone | - Tick if reported |
| Caregivers (target population includes caregivers) | - Tick if reported |
| Parental status | - Working parents - Single parents - Unspecified |
| Needs | - Social and emotional needs (social connections) - Purpose in life (civic engagement, meaningfulness and status, being able to contribute to society) - Mobility - Personal care needs - Meals - Domestic assistance - Accommodation (housing/home modifications and maintenance) - Financial management - Communication (language support/interpreters, information and assistance/referral services) - Skills development - Learning (e.g., new activity, new language, or learning about social skills) - Care navigation support or task orientation - Clinical or health needs - Respite care - Caregiver support |
| Setting | Where the intervention is being administered   - Personal home - Independent living (residential home) - Assisted living - Long-term care/ nursing home - Orphanage - School - Workplace - Community center, park, etc. - Art gallery or museum - Medical facility - Unspecified |
| WHO regions | - African Region - The Americas - South-East Asia Region - European Region - Eastern Mediterranean Region - Western Pacific Region - Multiple - Unspecified (any country is eligible) |
| World Bank income classification | - Low-income economies - Lower-middle income economies - Upper-middle income economies - High-income economies - Unspecified (any country is eligible) |
| Countries | - Africa (where multiple countries) - Europe (where multiple countries) - Latin america (where multiple countries) - Afghanistan - Albania - Algeira - Andorra - Angola - Antigua and Barbuda - Argentina - Armenia - Australia - Austria - Azerbaijan - Bahamas - Bahrain - Bangladesh - Barbados - Belarus - Belgium - Belize - Benin - Bhutan - Bolivia - Bosnia and Herzegovina - Botswana - Brazil - Brunei Darussalam - Bulgaria - Burkina Faso - Burundi - Cabo Verde - Cambodia - Cameroon - Canada - Central African Republic - Chad - Chile - China - Colombia - Comoros - Congo - Cook Islands - Costa Rica - Côte d’Ivoire - Croatia - Cuba - Cyprus - Czechia - Democratic People’s Republic of Korea - Democratic Republic of Congo - Denmark - Djibouti - Dominica - Dominican Republic - Ecuador - Egypt - El Salvador - Equatorial Guinea - Eritrea - Estonia - Eswatini - Ethiopia - Faore Islands - Fiji - Finland - France - Gabon - Gambia - Georgia - Germany - Ghana - Greece - Grenada - Guatemala - Guinea - Guinea-Bissau’ - Gayana - Haiti - Honduras - Hungary - Iceland - India - Indonesia - Iran - Iraq - Ireland - Israel - Italy - Jamaica - Japan - Jordan - Kazakhstan - Kenya - Kiribati - Kuwait - Kyrgyzstan - Lao People’s Democratic Republic - Latvia - Lebanon - Lesotho - Liberia - Libya - Lithuania - Luxembourg - Madagascar - Malawi - Malaysia - Maldives - Mali - Malta - Marshall Islands - Mauritania - Mauritius - Mexico - Micronesia - Monaco - Mongolia - Montenegro - Morocco - Mozambique - Myanmar - Namibia - Nauru - Nepal - Netherlands - New Zealand - Nicaragua - Niger - Nigeria - Niue - North Macedonia - Norway - Oman - Pakistan - Panama - Papua New Guinea - Paraguay - Peru - Philippines - Poland - Portugal - Puerto Rico - Qatar - Republic of Korea - Republic of Moldovia - Romania - Russian Federation - Rwanda - Saint Kitts and Nevis - Saint Lucia - Saint Vincent and the Grenadines - Samoa - San Marino - Sao Tome and Principe - Saudi Arabia - Senegal - Serbia - Seychelles - Sierra Leone - Singapore - Slovakia - Slovenia - Solomon Islands - Somalia - South Africa - South Sudan - Spain - Sri Lanka - Sudan - Suriname - Sweden - Switzerland - Syrian Arab Republic - Tajikistan - Thailand - Timor-Leste - Togo - Tokelau - Toonga - Trinidad and Tobago - Tunisia - Türkiye - turkmenistan - Tuvalu - Uganda - Ukraine - United Arab Emirates - United Kingdom of Great Britain and Northern Ireland - United Republic of Tanzania - United States of America - Uruguay - Uzbekistan - Vanuatu - Venezuela - Viet Nam - Yemen - Zambia - Zimbabwe |
| Equity analysis | |
| Does the study assess any differences in effects (benefit or harm) across any PROGRESS-Plus factors | - Place of residence (rural or remote/urban) - Race/Ethnicity/culture/language - Occupation - Gender or Sex - Religion - Education - Socioeconomic status - Social capital (e.g., marital status – widowed, divorced, separated) - Plus-factor – social isolated or at risk - Plus-factor – lonely or at risk - Plus-factor – age (older adults, children) - Plus-factor – disability - Plus-factor – frailty - Plus-factor – health status (e.g., dementia, disease severity) - Plus-factor – living situation (e.g., alone, long term care, away from home) |
